# Gender differences in attitudes and attributes of people using therapeutic shoes for diabetic foot complications

**DOI:** 10.1186/s13047-019-0327-0

**Published:** 2019-03-29

**Authors:** Gustav Jarl, John Alnemo, Roy Tranberg, Lars-Olov Lundqvist

**Affiliations:** 1 Department of Prosthetics and Orthotics, Faculty of Medicine and Health, Örebro University, SE 70182 Örebro, Sweden; 2 University Health Care Research Center, Faculty of Medicine and Health, Örebro University, SE 70182 Örebro, Sweden; 3 Department of Prosthetics and Orthotics, Sahlgrenska University Hospital, Gothenburg, Sweden; 4 Department of Orthopaedics, Institute of Clinical Sciences, the Sahlgrenska Academy at University of Gothenburg, Gothenburg, Sweden

**Keywords:** Diabetes mellitus, Diabetic foot, Diabetes complications, Shoes, Patient compliance, Treatment adherence and compliance

## Abstract

**Background:**

Therapeutic shoes can prevent diabetic foot reulcerations but their use is complicated by the fact that shoes have psychological and social meanings, which is believed to put a larger burden on women than men. The aim was to compare attitudes and attributes of women and men using therapeutic shoes for diabetic foot complications.

**Methods:**

A questionnaire was posted to 1230 people with diabetes who had been fitted with therapeutic shoes. Women’s and men’s answers were compared using *t*-tests, Mann–Whitney *U* tests and chi-square tests with Fischer’s exact tests. *P*-values < 0.05 were considered statistically significant.

**Results:**

Questionnaires from 443 (36.0%) respondents (294 men, 149 women, mean age 69.2 years) were analyzed. More men than women (*p* < 0.05) had paid employment (20.4% vs 9.4%), had someone who reminded them to wear their therapeutic shoes (27.6% vs 10.0%), and had a history of foot ulcers (62.9% vs 46.3%) or minor amputation (17.7% vs 6.7%). More women than men received disability pension (18.8% vs 10.2%). Women reported worse general health, lower internal locus of control regarding ulcer prevention, and more negative attitudes to the appearance and price of therapeutic shoes and how they felt about wearing them in public. Other comparisons were non-significant: other shoe attributes, education, diabetes type, current foot ulcers, major amputations, satisfaction with shoe services, understanding of neuropathy as a risk factor, locus of control regarding ulcer healing, belief in the shoes’ efficacy to prevent and heal ulcers, worries about ulcer healing and new ulcerations, self-efficacy, depression, shoe use/adherence, paying a fee for therapeutic shoes, and social support.

**Conclusions:**

Men had worse foot complications. Women had worse general health, lower internal locus of control regarding ulcer prevention, and more negative attitudes toward therapeutic shoes. Clinicians should pay more attention to their female patients’ concerns. Future research and development should focus on improving the weight and appearance of therapeutic shoes, particularly for women. Research is also needed on how to facilitate the adaption and reevaluation process where patients change from viewing shoes purely as items of clothing to also viewing them as medical interventions.

## Background

Diabetic foot ulcers are a common and devastating complication of diabetes. The lifetime incidence of foot ulcers has been estimated to be between 19 and 34%, and these ulcers are associated with significant morbidity, mortality, and risk of lower extremity amputations [1]. Although most ulcers heal, recurrence rates are high: approximately 40% of foot ulcers recur within 1 year of healing, and 60% within 3 years [1]. A systematic review [2] found that therapeutic shoes are beneficial in preventing the recurrence of plantar foot ulcers and, consequently, they are recommended in international guidelines [3]. Despite this, many patients use their therapeutic shoes less than recommended [4], which reduces the protective effect [5]. At first glance, the use of therapeutic shoes may seem trivial: if offloading is effective and adherence high, fewer foot ulcers will recur. However, using therapeutic shoes as medical interventions is complicated by the fact that shoes have psychological and cultural meanings that transcend their functionality [6]. The transformation from the everyday perspective of shoes as items of clothing to a medical perspective of shoes as medical interventions is challenging and can take considerable time and effort [7]. Qualitative studies on people with diabetes have suggested that women in particular dislike wearing therapeutic shoes, which they view as big, ugly, and unfeminine, affecting their self-image [7–9]. However, most quantitative studies on gender differences in people with diabetic foot complications have more narrowly focused on adherence to wearing therapeutic shoes, and a review from 2016 [10] found no gender difference in adherence in the five included studies [4, 11–14]. One quantitative study [15] investigated gender differences in attitudes toward therapeutic shoes and found that, although fewer of the men were given a choice of shoe style, they were still more satisfied with the shoes than the women, suggesting that future quantitative studies should investigate gender aspects of therapeutic shoe use from a wider perspective. Thus, the aim of this study was to compare attitudes and attributes of women and men using therapeutic shoes for diabetic foot complications.

## Methods

### Study design and setting

The study design was cross-sectional and observational, using a postal survey that was sent to patients at two prosthetic and orthotic clinics in two counties in Sweden.

### Construction and pilot test of questionnaire

A questionnaire was constructed based on a literature review of factors that affect adherence to wearing therapeutic shoes among people with diabetes [10]. Factors from the health belief model [16] were also included in the questionnaire. The questions covered demographics, diabetes and foot complications, satisfaction with services, understanding of sensory neuropathy as a risk factor for foot ulcers, internal locus of control, belief in efficacy of therapeutic shoes, worries about prevention and healing of foot ulcers, self-efficacy, general health, depression, attitudes to therapeutic and conventional shoes, shoe use, reminders to use therapeutic shoes, and social support. Several of the items were copied or adapted from existing questionnaires [17–25].

A pilot test of the questionnaire was conducted on five individuals (two women, three men, median age 67 years, range 48–80 years) with diabetes and previous or current foot ulcers. They answered the questionnaire and were later interviewed by telephone by the first author about their understanding of the questions and response alternatives. Only minor adjustments to the questionnaire were needed as a result of the interviews: one question was reworded and a new response alternative was added to another question.

### Participants and procedure

The inclusion criteria were having diabetes, being at least 18 years of age (on January 1, 2016), having been prescribed therapeutic shoes at some point in time, and having visited one of two prosthetic and orthotic clinics in the period January to December 2016. The exclusion criterion was bilateral major amputation. The questionnaire and study information were sent by post to the 1230 people fulfilling these criteria and still living in May and June 2017. A reminder was sent 1 month later to those who had not returned the questionnaire.

### Statistical analyses

Women’s and men’s answers were compared using two-sided *t*-tests, Mann–Whitney *U* tests, and chi-square tests with Fischer’s exact tests. *P*-values less than 0.05 were considered statically significant. IBM SPSS, version 22, was used for the statistical analyses.

## Results

Of the 1230 people who received the questionnaire, 469 returned valid answers and, of those, 26 were excluded because the respondents stated that they did not own any therapeutic shoes. The remaining 443 questionnaires were included in the analysis, giving a response rate of 36.0%. The majority of the respondents were retired men, with type 2 diabetes (Table 1). Comparisons of demographic and disease-related variables revealed that more men than women were in paid employment and had a history of foot ulcers or minor amputations. More women than men reported that they received disability pension. There were no significant gender differences in age, education, diabetes type, current foot ulcers, or major amputations.

**Table 1:** Demographic characteristics of participants

		All (*n* = 443)	Men (*n* = 294)	Women (*n* = 149)	*P*-value^a^
Age, mean (sd)		69.2 (10.6)	69.1 (10.3)	69.4 (11.4)	0.794
Education, n (%)	Incomplete elementary schooling	11 (2.5)	8 (2.7)	3 (2.0)	0.758^b^
	Elementary school	150 (33.9)	103 (35.0)	47 (31.5)	0.502
	Upper secondary school	154 (34.8)	104 (35.4)	50 (33.6)	0.756
	College/university	110 (24.8)	68 (23.1)	42 (28.2)	0.218
	Missing	18 (4.1)	11 (3.7)	7 (4.7)	
Occupation, n (%)^c^	Retired	306 (69.1)	206 (70.1)	100 (67.1)	0.548
	Paid employment	74 (16.7)	60 (20.4)	14 (9.4)	**0.003**
	Unemployed	12 (2.7)	7 (2.4)	5 (3.4)	0.547^b^
	Student	3 (0.7)	1 (0.3)	2 (1.3)	0.262^b^
	Disability pension	58 (13.1)	30 (10.2)	28 (18.8)	**0.011**
	Sick leave	25 (5.6)	16 (5.4)	9 (6.0)	0.790
	Missing	8 (1.8)	5 (1.7)	3 (2.0)	
Diabetes type, n (%)	Type 1	120 (27.1)	71 (24.1)	49 (32.9)	0.051
	Type 2	320 (72.2)	220 (74.8)	100 (67.1)	0.087
	Other type	3 (0.7)	3 (1.0)	0	0.554^b^
	Missing	0	0	0	
Current ulcer, n (%)	Yes	135 (30.5)	89 (30.3)	46 (30.9)	0.897
	No	305 (68.8)	203 (69.0)	102 (68.5)	
	Missing	3 (0.7)	2 (0.7)	1 (0.7)	
Previous ulcer, n (%)	Yes	254 (57.3)	185 (62.9)	69 (46.3)	**0.001**
	No	175 (39.5)	100 (34.0)	75 (50.3)	
	Missing	14 (3.2)	9 (3.1)	5 (3.4)	
Minor amputation, n (%)	Yes	62 (14.0)	52 (17.7)	10 (6.7)	**0.002**
	No	370 (83.5)	237 (80.6)	133 (89.3)	
	Missing	11 (2.5)	5 (1.7)	6 (4.0)	
Major amputation, n (%)	Yes	20 (4.5)	17 (5.8)	3 (2.0)	0.074
	No	414 (93.5)	272 (92.5)	142 (95.3)	
	Missing	9 (2.0)	5 (1.7)	4 (2.7)	

^a^A two-sided *t*-test was used to compare the age of men and women. Two-sided chi-square tests were used for all other comparisons. For variables with two response categories, a single chi-square test was used. For variables with three or more response categories, a separate chi-square test was used for each response category. *P*-values less than 0.05 are written in boldface

^b^Two-sided Fischer’s exact test was used because at least one cell had an expected cell count below 5

^c^The percentages add up to more than 100% because more than one alternative could be chosen

Both women and men were satisfied with the shoe services at the prosthetic and orthotic clinic, agreed that sensory neuropathy increases the risk of foot ulcers, expressed internal locus of control for prevention and healing of foot ulcers, agreed that using therapeutic shoes can improve prevention and healing, were somewhat worried about prevention and healing, and expressed self-efficacy about using therapeutic shoes (Table 2). Compared to men, women reported worse general health and lower internal locus of control regarding prevention of ulcerations.

**Table 2:** Summary of responses to questions about attitudes and beliefs (abbreviated item texts), mean values (SD)

	All	Men	Women	*P*-value^a^
1. The staff were responsive to my concerns and questions	1.5 (0.6)	1.5 (0.6)	1.6 (0.6)	0.146
2. I was a partner in the decision-making with clinic staff	1.6 (0.6)	1.6 (0.6)	1.6 (0.6)	0.740
3. I am satisfied with the follow-up of my therapeutic shoes	2.2 (1.2)	2.2 (1.2)	2.2 (1.2)	0.809
4. Lost/reduced sensation in your feet increases the risk of foot ulcerations	2.1 (1.2)	2.1 (1.2)	2.1 (1.3)	0.988
5. What you do yourself is the main thing that affects whether your foot ulcer heals	2.1 (0.9)	2.1 (0.9)	2.1 (0.9)	0.860
6. What you do yourself is the main thing that affects whether you develop new foot ulcers	2.3 (1.1)	2.2 (1.1)	2.5 (1.2)	**0.048**
7. Probability of ulcer healing within 3 months if I always use therapeutic shoes	2.1 (0.9)	2.1 (0.9)	2.3 (0.9)	0.155
8. Probability of ulcer healing within 3 months if I never use therapeutic shoes	2.8 (1.0)	2.9 (1.0)	2.8 (1.0)	0.671
9. Probability of new ulceration within 12 months if I always use therapeutic shoes	3.0 (0.8)	3.0 (0.8)	2.9 (0.9)	0.439
10. Probability of new ulceration within 12 months if I never use therapeutic shoes	2.2 (1.0)	2.3 (1.0)	2.1 (1.0)	0.101
11. Worried that my foot ulcer(s) will never heal	2.7 (1.2)	2.8 (1.2)	2.6 (1.3)	0.271
12. Worried about getting new foot ulcers in the future	3.0 (1.3)	3.0 (1.3)	3.1 (1.4)	0.641
13. Confident I would always use therapeutic shoes if I decided to do so	1.9 (1.0)	1.9 (0.9)	2.0 (1.2)	0.611
14. General health	3.4 (1.0)	3.4 (1.0)	3.6 (0.9)	**0.014**
15. Feeling down, depressed, or hopeless	3.2 (1.0)	3.3 (1.0)	3.2 (1.0)	0.297

^a^Two-sided Mann–Whitney U test. The *p*-values less than 0.05 are written in boldface. Rating scales, items 1–3: 1 = strongly agree to 3 = disagree; items 4–6: 1 = strongly agree to 5 = strongly disagree; items 7–10: 1 = Highly probable to 4 = highly improbable; items 11–12: 1 = Very much to 5 = Not at all; item 13: 1 = Very certain to 4 = Very uncertain; item 14: 1 = Excellent to 5 = Bad, item 15: 1 = Almost every day to 4 = Not at all

In the sample as a whole, therapeutic shoes were preferred to conventional shoes when it came to the shoes’ effect on preventing and healing foot ulcers, price, fit, difficulties putting them on and taking them off, pain when standing or walking, difficulties walking in them, and using them in daily activities (Table 3). On the other hand, they preferred conventional shoes to therapeutic shoes when it came to the appearance and weight of the shoes. When comparing women and men, women held more negative attitudes to the appearance and price of therapeutic shoes, and preferred to wear conventional shoes in public. More men than women reported that clinic staff and people close to them reminded them to use their therapeutic shoes (Table 4). There were no gender differences in time spent wearing therapeutic and conventional shoes, paying a fee for therapeutic shoes, or the availability of social support from a relative or friend.

**Table 3:** Preferences for therapeutic and conventional shoes, mean values (SD)

	All	Men	Women	*P*-value^a^
Effect on ulcer healing	1.8 (0.8)	1.8 (0.8)	1.9 (0.9)	0.357
Effect on reducing risk of new ulcers	1.8 (0.8)	1.8 (0.8)	1.8 (0.8)	0.588
Difficulties walking in the shoes	2.3 (1.1)	2.3 (1.1)	2.3 (1.2)	0.958
Appearance	3.4 (1.2)	3.3 (1.2)	3.6 (1.2)	**0.006**
Weight	3.3 (1.1)	3.3 (1.1)	3.3 (1.1)	0.563
Price	2.7 (1.1)	2.5 (1.1)	2.9 (1.1)	**0.003**
Pain when standing and walking	2.1 (1.0)	2.1 (0.9)	2.2 (1.1)	0.216
Difficulties putting on and taking off the shoes	2.6 (1.0)	2.5 (1.0)	2.7 (1.0)	0.063
Ease of use in everyday activities, e.g. in your work	2.3 (1.1)	2.3 (1.1)	2.3 (1.1)	0.982
Feeling inclined to wear the shoes in public	3.0 (1.1)	2.9 (1.1)	3.2 (1.1)	**0.024**
Fit of the shoes	2.2 (1.1)	2.2 (1.0)	2.3 (1.1)	0.517

^a^Two-sided Mann–Whitney U test. *P*-values less than 0.05 are written in boldface. Rating scale: 1 = therapeutic shoes are much better, 2 = therapeutic shoes are better, 3 = no difference, 4 = conventional shoes are better, 5 = conventional shoes are much better

**Table 4:** Shoe use, fees, and social support

	All (*n* = 443)	Men (*n* = 294)	Women (*n* = 149)	*P*-value^a^
Weekly use of therapeutic shoes, days/week (SD)	5.9 (2.0)	5.9 (2.0)	5.8 (2.0)	0.691
Weekly use of conventional shoes, days/week (SD)	2.2 (2.8)	2.0 (2.7)	2.4 (2.9)	0.198
Daily use of therapeutic shoes, hours/day (SD)	8.5 (4.7)	8.3 (4.6)	8.9 (4.9)	0.214
Daily use of conventional shoes, hours/day (SD)	3.4 (3.3)	3.4 (3.4)	3.4 (3.2)	0.991
Percentage of waking day in therapeutic shoes, mean (SD)	50.3 (32.8)	49.4 (32.0)	52.2 (34.5)	0.395
Percentage of waking day in conventional shoes, mean (SD)	12.1 (21.1)	11.5 (21.1)	13.3 (21.3)	0.419
Use of therapeutic shoes at least 60% of waking day, n (%)				
Yes	169 (38.1)	110 (37.4)	59 (39.6)	0.520
No	260 (58.7)	177 (60.2)	83 (55.7)	
Missing	14 (3.2)	7 (2.4)	7 (4.7)	
Did you pay a fee for your current therapeutic shoes? n (%)				
Yes	335 (75.6)	218 (74.1)	117 (78.5)	0.420
No	92 (20.8)	64 (21.8)	28 (18.8)	
Missing	16 (3.6)	12 (4.1)	4 (2.7)	
Does someone usually remind you to use your therapeutic shoes?, n (%)^b^				
Yes, clinic staff	58 (13.1)	47 (16.0)	11 (7.4)	**0.014**
Yes, people close to me	50 (11.3)	44 (15.0)	6 (4.0)	**0.001**
No	331 (74.7)	205 (69.7)	126 (84.6)	**< 0.001**
Missing	16 (3.6)	8 (2.7)	8 (5.4)	
Is there someone close to you who supports you with your foot problems? n (%)				
Yes	262 (59.1)	178 (60.5)	84 (56.4)	0.363
No	168 (37.9)	107 (36.4)	61 (40.9)	
Missing	13 (2.9)	9 (3.1)	4 (2.7)	

^a^Two-sided t-tests were used for comparisons of weekly and daily shoe use. For variables with two response categories, a single chi-square test was used. For variables with three or more response categories, a separate chi-square test was used for each response category. *P*-values less than 0.05 are written in boldface. Adherence was estimated with two questions adapted from the Questionnaire for persons with a transfemoral amputation [19]

^b^The percentages add up to more than 100% because some respondents were reminded by both clinic staff and people close to them (e.g. a relative or friend)

## Discussion

The aim of this study was to compare attitudes and attributes of women and men using therapeutic shoes. Like many other studies on people with diabetic foot complications, the majority of our respondents were men. Somewhat surprisingly, men reported more severe foot complications than women, but more men than women were in paid employment and fewer were on disability pension. This might be related to the fact that women had worse general health, but the cross-sectional and observational study design precludes any inferences about the casual relationships. Men to a greater extent reported that clinic staff and people close to them reminded them to wear their therapeutic shoes. This is in accordance with our clinical experience that the wives of many male patients are actively involved in the care of their husbands, while the husbands of female patients may not be involved to the same extent. More troubling is the fact that the female patients were reminded less by the clinic staff, which suggests that female and male patients are to some extent treated differently.

Both women and men preferred therapeutic shoes when it came to their functional aspects. This is in accordance with other studies, in which users of therapeutic shoes have generally been satisfied with the fit [13, 15] and comfort [4, 12, 15] and have felt that therapeutic shoes were of benefit to their feet [15]. In contrast, both women and men preferred the appearance and weight of conventional shoes, and the preference for their appearance was more pronounced among the women. The higher dissatisfaction among women with the appearance of therapeutic shoes and their disinclination to wear them in public may be related to the fact that fashionable shoes for women are often less functional than fashionable shoes for men; gender differences in shoe styles are large [26] although gender differences in foot shape are relatively small when adjusted for foot length [27]. Many studies have reported low satisfaction with and complaints about the appearance [4, 12, 13, 28] and weight [12, 28] of therapeutic shoes, but usually without comparing women’s answers with men’s, which may have obscured gender differences like the ones that we found. Still, the more negative attitudes of women toward the appearance of therapeutic shoes and how they felt about wearing them in public are in accordance with qualitative studies, in which women have stressed how unattractive and unfeminine therapeutic shoes are and how this impacts their self-esteem and participation in social life [7–9, 29]. In our study, women had less positive opinions about the price they paid for their therapeutic shoes, although the proportion of women and men that had paid a fee for their shoes did not differ. The explanation may be that women were more dissatisfied with other aspects of their therapeutic shoes, making them feel that the therapeutic shoes were not worth the fee paid for them. It is less clear why women reported lower internal locus of control regarding prevention of ulcerations, especially as there was no gender difference in locus of control regarding ulcer healing.

Adherence to wearing therapeutic shoes have been investigated in several studies but comparisons with other studies are difficult because they often define and measure adherence in different ways [30]. However, two studies [12, 28] using a similar methodology to the current study, that is, questionnaires asking participants to estimate how much they wore their therapeutic shoes, report that 42% of the participants wore their therapeutic shoes for at least 60% of their waking day. This is similar to our study, where 38.1% wore their therapeutic shoes for at least 60% of their waking day. We found no association between gender and adherence, which is consistent with findings from previous studies [4, 11–14] and is interesting considering that the female respondents had more negative opinions about their therapeutic shoes. This suggests that adherence may be influenced by multiple factors [10], and will be investigated with multivariate analysis in future studies on this dataset.

Although we found some differences between women and men, we should not forget that most comparisons were non-significant. This could be related to the fact that the study was conducted in Sweden, which is ranked among the countries with the smallest gender gaps in the world [31]; the observed gender similarities may therefore not be transferable to countries with larger gender gaps. With this is mind, the observed results point to some implications for clinical practice and research. First, developments in shoe design and materials are needed to reduce the weight of therapeutic shoes. Second, the issue of the appearance of therapeutic shoes needs more attention, in particular for women’s shoes. There may be a need to make certain models of therapeutic shoes more feminine, without compromising their fit and function. Female patients are often in the minority, and it is important for clinicians to remind them to wear their therapeutic shoes and to be aware that their preferences may differ from those of the male patients. Also, clinicians and researchers need to pay more attention to how to facilitate the reevaluation process, in which patients change their views and priorities from only viewing shoes as items of clothing to also viewing them as medical interventions. As a result, they give weight not only to esthetic attributes but also to functional benefits such as preventing foot ulcerations.

### Limitations

This study has some limitations. First, the study sample consisted of patients who had received therapeutic shoes and thus patients who refused therapeutic shoes in the first place were not included. Thus, the study may have underestimated the negative attitudes toward therapeutic shoes. Second, the survey respondents were patients from two prosthetics and orthotics clinics in Sweden, and these patients may not be representative of all patients with diabetic foot complications. Third, the response rate was low and we cannot know whether the results of comparing women and men would be the same if all people surveyed had answered the questionnaire.

## Conclusions

Our major findings were that men had more severe foot complications than women but women had worse general health, lower internal locus of control regarding ulcer prevention, and more negative attitudes toward therapeutic shoes. Clinicians need to pay more attention to their female patients’ concerns. Future research and development should focus on improving the weight and appearance of therapeutic shoes, in particular shoes for women. Also, more research is needed on how to facilitate the adaption and reevaluation process in which patients change from viewing shoes purely as items of clothing to also viewing them as medical interventions.
